# Conformational Change of a Tryptophan Residue in BtuF Facilitates Binding and Transport of Cobinamide by the Vitamin B12 Transporter BtuCD-F

**DOI:** 10.1038/srep41575

**Published:** 2017-01-27

**Authors:** S. A. Mireku, M. Ruetz, T. Zhou, V. M. Korkhov, B. Kräutler, K. P. Locher

**Affiliations:** 1 Institute of Molecular Biology and Biophysics, Eidgenössische Technische Hochschule (ETH) Zürich, CH-8093 Zürich, Switzerland; 2 Institute of Organic Chemistry and Center of Molecular Biosciences, University of Innsbruck, Innrain 80/82, A-6020 Innsbruck, Austria

## Abstract

BtuCD-F is an ABC transporter that mediates cobalamin uptake into *Escherichia coli*. Early *in vivo* data suggested that BtuCD-F might also be involved in the uptake of cobinamide, a cobalamin precursor. However, neither was it demonstrated that BtuCD-F indeed transports cobinamide, nor was the structural basis of its recognition known. We synthesized radiolabeled cyano-cobinamide and demonstrated BtuCD-catalyzed *in vitro* transport, which was ATP- and BtuF-dependent. The crystal structure of cobinamide-bound BtuF revealed a conformational change of a tryptophan residue (W66) in the substrate binding cleft compared to the structure of cobalamin-bound BtuF. High-affinity binding of cobinamide was dependent on W66, because mutation to most other amino acids substantially reduced binding. The structures of three BtuF W66 mutants revealed that tight packing against bound cobinamide was only provided by tryptophan and phenylalanine, in line with the observed binding affinities. *In vitro* transport rates of cobinamide and cobalamin were not influenced by the substitutions of BtuF W66 under the experimental conditions, indicating that W66 has no critical role in the transport reaction. Our data present the molecular basis of the cobinamide versus cobalamin specificity of BtuCD-F and provide tools for *in vitro* cobinamide transport and binding assays.

ATP-binding cassette (ABC) transporters are a large family of integral membrane proteins and involved in nutrient uptake, drug extrusion, and lipid homeostasis. They use the energy of ATP binding and hydrolysis to power substrate transport across the lipid bilayer^1^,^2^. Depending on the direction of transport, into the cytoplasm or out, they are classified as importers or exporters, respectively. Prokaryotic importers depend on external substrate binding proteins (SBPs) for delivery of the cargo to the membrane-embedded transporters^3^. In the Gram-negative bacterium *E. coli*, BtuF is the periplasmic SBP that delivers cyanocobalamin (Cbl) to the ABC type II importer BtuCD^4^,^5^,^6^. BtuCD-F has been studied extensively by structural and functional techniques^7^,^8^,^9^ and a detailed transport mechanism has been formulated to rationalize the coupling of nucleotide binding and hydrolysis to conformational changes facilitating active transport^10^,^11^.

*E. coli* is unable to synthesize Cbl de novo. However, it expresses enzymes that facilitate its synthesis from the precursor cobinamide (Cbi)^12^, which lacks the 5,6-dimethylbenzimidazole (DMB) moiety and sugar-phosphate linker and is therefore smaller than Cbl (Figs 1A and 2C). Before the individual proteins (BtuC, BtuD, BtuF) were identified, *in vivo* transport data suggested that the *E. coli* uptake system specific for Cbl also transports Cbi^13^. In line with this observation, recent *in vitro* binding data showed that *Thermotoga* species BtuF variants can bind not only Cbl, but also Cbi^14^. The authors base their finding on changes in Tm values measured by differential scanning fluorimetry and shifts in the visible spectra upon ligand binding. They propose that *Thermotogales* species BtuCD-F transporters can import both Cbl as well as Cbi. Although the Cbl-bound BtuF structures revealed the molecular organization of the substrate binding site at high resolution^15^,^16^, it is not known whether and how *E. coli* BtuF would recognize and bind Cbi in addition to Cbl. Also, Cbi transport by BtuCD-F was never demonstrated.

We pursued structural and functional approaches to address these questions. We developed an *in vitro* transport assay and showed that BtuCD could indeed transport Cbi in an ATP- and BtuF-dependent manner. We also established an *in vitro* binding assay that allowed Cbi and Cbl binding to BtuF to be quantitated. We identified a single tryptophan residue (W66) in BtuF that adopts a distinct conformation depending on whether Cbi or Cbl is bound.

## Results

### ^14^CN-Cbi synthesis and *in vitro* transport

**Figure 1:**
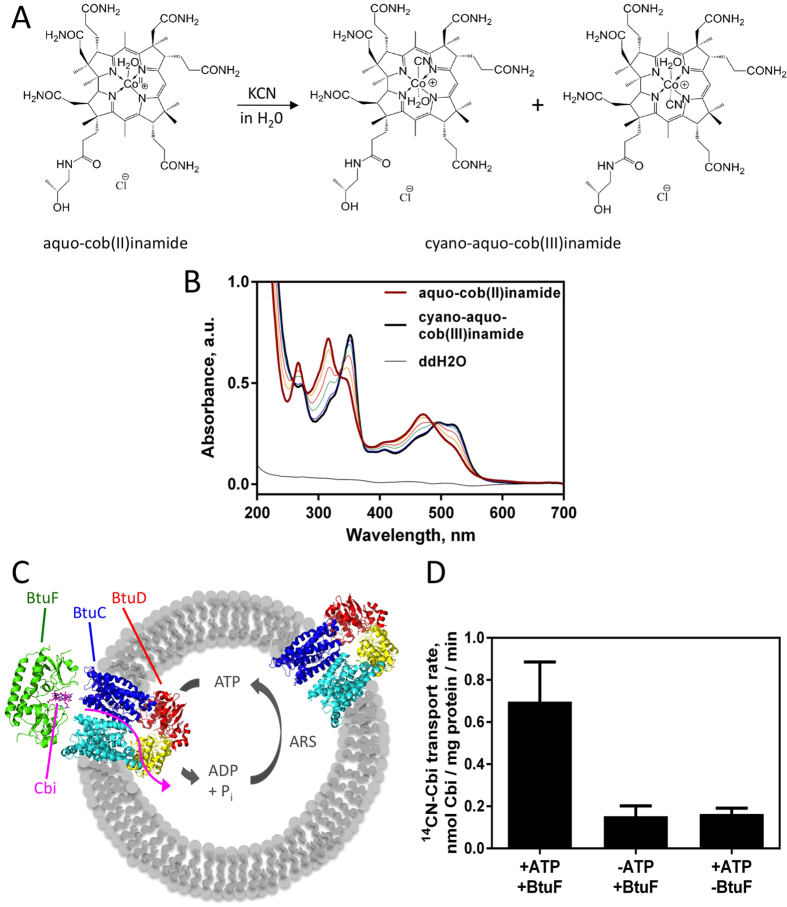
Generation of radioactive cobinamide and *in vitro* transport by BtuCD. (**A**) Reaction scheme of ^14^CN-Cbi generation (^14^C-labeled cyano-aquo-cob(III)inamide). Radiolabeled cobinamide was generated in an oxidative reaction by addition of ^14^C-labeled KCN to aquo-cob(II)inamide. The CN group in the product can be coordinated in the α- or β-position of the corrin ring. (**B**) UV/Vis spectra of ^14^CN-Cbi generation. A 0.85 mol equivalent of K^14^CN was titrated to 4.8 μmol aquo-cob(II)inamide at 22 °C. UV/Vis spectra of the reaction mix diluted to 47 μM cobinamide were recorded 30 to 60 min after each sequential addition of K^14^CN and ddH_2_O was used for the blank measurement. The final product cyano-aquo-cobinamide is represented with the black line and the educt aquo-cob(II)inamide is shown in brown. Titration steps in between are shown with thin lines. (**C**) Schematic of *in vitro* Cbi transport by BtuCD reconstituted into liposomes. Only the right-side-in oriented BtuCD molecules are accessible for BtuF and yield successful transport. The direction of substrate transport into the liposome lumen is indicated with a pink arrow. To keep a constant ATP concentration within the time course of the experiment, an ATP-regenerating system (A.R.S.) was incorporated into the liposome lumen. (**D**) Cbi transport activity of wild type BtuCD-F. Transport was measured under the following conditions: 4 mg/ml proteoliposomes (~0.5 μM total BtuCD), 1 μM BtuF, 15 μM cobinamide and 2 mM ATP. Unspecific radioligand binding to liposomes was determined by omission of ATP or BtuF. Shown are mean ± S.E.M. (n = 6) of initial transport rates. For lines used to determine initial rates see Supplementary Figure 2.

To investigate *in vitro* Cbi transport by BtuCD, radiolabeled substrate was required, which is not commercially available. We exploited the fact that CN-Cbi (cyano-aquo-cobinamide) is a stable and inert species and synthesized ^14^CN-Cbi by an oxidative reaction using aquo-cob(II)inamide and ^14^C-labeled KCN as educts (Fig. 1A). A 0.85 mol equivalent of K^14^CN was titrated to aquo-cob(II)inamide, since a 1:1 ratio already leads to the formation of a small fraction of di-cyano-cobinamide. The ^14^CN-Cbi product formation was monitored by recording UV/Vis spectra, which clearly showed shifts of the absorption peaks in the range of 300–400 nm and 450–550 nm (Fig. 1B). Formation of di-cyano-cobinamide could be ruled out by the absence of an absorption peak at 580 nm (Suppl. Figure 1). In addition, we analyzed the reaction mix using silica thin layer chromatography, because educt and product can be separated by this method (data not shown).

For transport assays, BtuCD was reconstituted into liposomes. Because only transporters oriented right-side-in (BtuD in the liposome lumen) can catalyze productive substrate transport (Fig. 1C), we determined the right-side-in reconstitution efficiency using a fluorescein-labeled BtuF variant (BtuF_fluo_) that was previously shown to be fully functional^11^. We determined that ~10% of BtuCD could associate with BtuF_fluo_ and thus were oriented right-side-in (data not shown). This value agrees with the previously determined 7 ± 2%, which was established by measuring the amount of trapped, non-transported, radio-labeled Cbl associated with BtuCD^7^. To start the Cbi transport reaction, BtuF previously incubated with ^14^CN-Cbi was added to BtuCD-containing liposomes. All transport rates were corrected for the amount of right-side-in oriented transporters and later also for the background level of Cbi associating with proteoliposomes in the absence of ATP. We determined an initial transport rate of 0.7 ± 0.2 nmol Cbi/mg protein/min (Fig. 1D and Suppl. Figure 2), with a 2-fold variability in absolute rates depending on the proteoliposome batch. BtuCD-catalyzed Cbi transport was ATP- and BtuF-dependent, as was observed for Cbl transport^7^. The calculated *in vitro* turnover number for Cbi transport was approximately 1.5-fold slower than that of Cbl transport. This makes Cbi a bona fide substrate of *E. coli* BtuCD-F.

### Crystal structure of Cbi-bound BtuF

**Figure 2:**
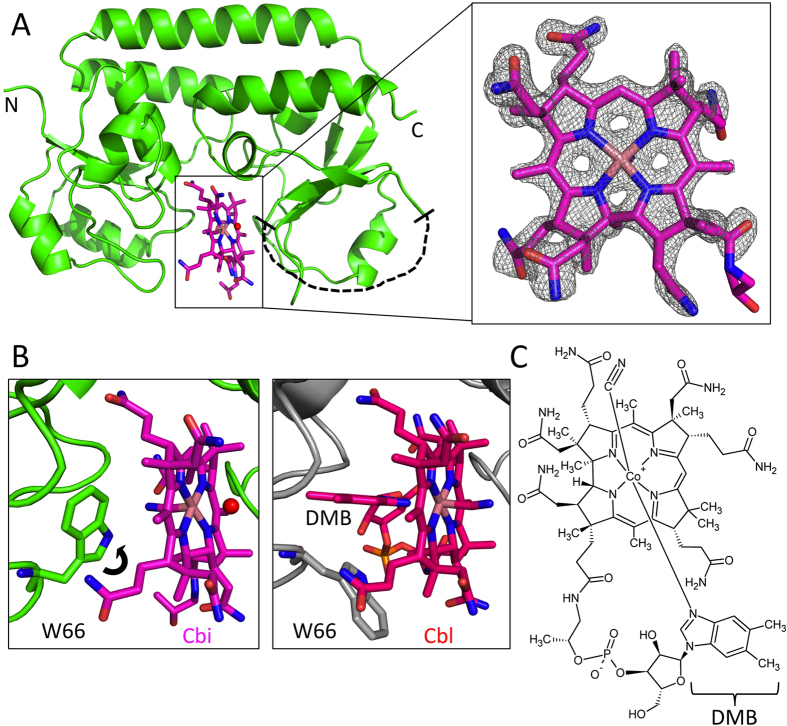
Crystal structure of Cbi-bound BtuF. (**A**) Cbi-bound BtuF model (PDB ID 5M29). BtuF is depicted in green cartoon and cyano-aquo-cobinamide is shown in stick representation (carbon, mangenta; oxygen, red; nitrogen, blue; cobalt, light brown). The N- and C-termini of BtuF are indicated as N and C, respectively. After initial rounds of refinement with omission of the ligand in the model, unambiguous electron density for Cbi was displayed in the Fo-Fc map contoured at ±3.0σ (box to the right and viewed from C-lobe). The disordered amino acid residue stretch (217 to 232) is indicated with a dashed black line. (**B**) Comparison of the substrate binding sites of Cbi-bound BtuF (left box) and Cbl-bound BtuF (right box, PDB ID 1N4A). In Cbi-bound BtuF residue W66 adopts a new rotamer (black arrow) facing the inside of the substrate binding site and filling the space occupied by DMB in Cbl-bound BtuF. The two axial ligands of Cbi, a cyanide and a water molecule, are shown as stick and red sphere, respectively. For Cbi-bound BtuF the same coloring was used as in panel A. Cbl-bound BtuF is illustrated as gray cartoon for the protein part and stick representation for Cbl (carbon, red; oxygen, red; nitrogen, blue; cobalt, light brown; phosphate, yellow). Both substrate binding sites are shown from the front view similar to panel A. (**C**) Chemical structure of the bigger substrate Cbl illustrating the additional 5,6-dimethylbenzimidazole (DMB) moiety and sugar-phosphate linker, which are absent in the smaller substrate Cbi. The two axial ligands DMB and cyanide are in the α- and β-position, respectively, and coordinated by the central Co atom.

The crystal structure of mono-cyano-cobinamide bound BtuF (CN-Cbi-BtuF) was determined at 1.5 Å resolution by molecular replacement using Cbl-bound BtuF (PDB ID 1N2Z) devoid of Cbl as a model (Fig. 2). Well-defined electron density in the difference map was observed for bound Cbi as well as the side chain of W66 after initial rigid-body refinement using BtuF devoid of substrate (Fig. 2A). The structure of Cbi-bound BtuF was similar to that of Cbl-bound BtuF (pdb ID 1N4A) with a RMSD of 0.419 Å when using the corrin ring to anchor the superposition. The six residues Y50, W66, W85, F162, F168 and W196 in the substrate cavity all contribute to Cbi binding, which was previously reported for Cbl-bound BtuF^15^,^16^. The only significant structural difference was observed for W66, whose side chain swung approximately 130° around the Cα-Cβ bond towards the inside of the binding pocket, to the location that is occupied by the 5-methyl group of DMB in Cbl-bound BtuF (Fig. 2B and C). There is no direct coordination of the Co atom of Cbi by the side chain of W66, unlike the bond between the DMB moiety and the Co atom in Cbl-bound BtuF^15^. Instead, we observed density for the CN group bound to the Co atom, facing the W66 side chain. Residues 217–232 in Cbi-bound BtuF were not visible in the electron density, indicating flexibility in this region, whereas this stretch forms an α-helix in Cbl-bound BtuF. It is not clear whether this is a crystallographic artefact or of biological relevance. Similarly high flexibility in this region was observed for the apo-structure of BtuF^16^.

### Binding of Cbi and Cbl to BtuF W66 mutants

**Figure 3:**
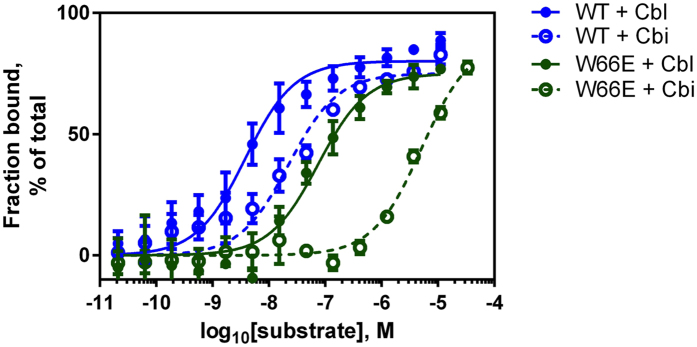
Cbl and Cbi binding to wild type BtuF_fluo_ and W66E. The substrate binding assay is based on the quenching of the fluorescence signal of fluorescein labeled BtuF (BtuF_fluo_) in a substrate concentration dependent manner. BtuF_fluo_ was used at 5 nM and the substrate concentration was ranged from 0.02 nM to 33 μM. Shown is one experiment performed in triplicates and indicated are mean and SD (n = 3). For determined K_D_ values see Table 1. Same experiment as shown in Supplementary Figure 4.

To investigate the role of W66 in substrate binding and transport, we generated various W66 mutations and analyzed *in vitro* substrate binding with a fluorescence based binding assay. The binding assay was performed with CN-Cbl or di-CN-Cbi and BtuF_fluo_ (Suppl. Figure. 3). We found that binding of Cbl or Cbi quenches the fluorescence of the covalently attached fluorescein label, allowing the precise determination of bound substrate. Using this assay, we determined a dissociation constant K_*D*_ of Cbl and wt BtuF_fluo_ in the low nM range, as reported previously^5^. No substantial changes in affinity for Cbl could be determined for W66 mutants (Fig. 3 and Table 1) except for mutations to charged side chains (arginine or glutamate), which caused a decrease in affinity of ~7-fold. In contrast, binding of Cbi to BtuF was strongly affected by mutations of W66. The K_*D*_ of wt BtuF for Cbi was 40 ± 10 nM, and the affinity of the W66X mutants was lower except for W66F (30 ± 7 nM, Table 1). Substitution of W66 with tyrosine or leucine reduced the affinity 3-fold compared to wt, and a change to histidine or arginine reduced it more than 10-fold (Table 1). The largest change was observed for the W66E and W66A mutations, with K_*D*_ values in the low micromolar range. These results indicate that high affinity Cbi binding requires an aromatic, hydrophobic, and bulky residue such as tryptophan or phenylalanine at position 66 for strong Cbi binding. [Table t2]

### Crystal structures of Cbi-bound BtuF mutants W66F, W66Y and W66L

**Figure 4:**
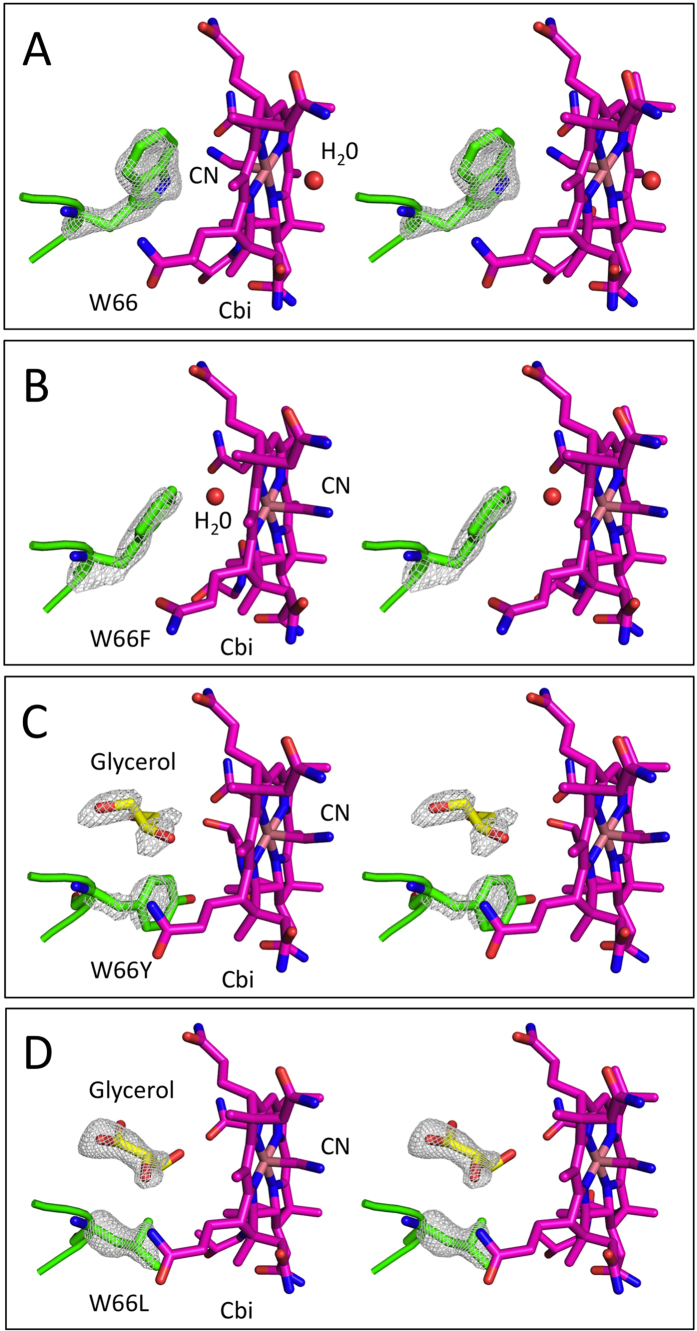
Stereo view of the substrate binding sites of Cbi-bound BtuF mutants in comparison to the wild type. Displayed are residue W66 in wild type BtuF (**A**) and the corresponding mutants W66F (**B**), W66Y (**C**) and W66L (**D**). Cobinamide was bound in all 4 crystal structures and was used as reference point for model alignment. In W66F the phenylalanine rotamer is as in the wild type structure facing the inside of the cavity. In W66Y as well as in W66L, the side chain is facing outside of the cavity. A glycerol molecule shown in yellow is occupying the space instead. Glycerol was used during purification and for cryoprotection, but was not present in the crystallization condition. The position of bound cyanide varied between the two protein chains in the asymmetric unit. For W66, W66F and W66Y chain A is shown and for W66L chain B. For the mutants W66Y and W66L only the cyanide group is shown, because no electron density was visible for the second axial ligand. The two potential axial ligands, cyanide and a water molecule, are represented in stick and red sphere, respectively. CN-Cbi is illustrated as stick representation (carbon, mangenta; nitrogen, blue; oxygen, red; Co, light brown). Displayed is the difference density of an omit map (Fo-Fc contoured at ±3.0σ) calculated by Phenix and using a model in which the occupancy of the side chain and Cα were set to zero. See result section for more detailed description. PDB IDs of the models are 5M29 (BtuF-Cbi), 5M2Q (BtuF_W66F_-Cbi), 5M34 (BtuF_W66Y_-Cbi) and 5M3B (BtuF_W66L_-Cbi).

To visualize the structural basis of the observed functional effects of W66 mutations, crystal structures of Cbi-bound BtuF containing the mutations W66F, W66Y or W66L were determined at similar resolutions as the wild type construct (Table 2). The refined wild type model with mutated W66A and deleted water molecules was used as a search model for molecular replacement. The structures of the mutants were similar to that of the wild type, and differences were only observed in the substrate-binding pocket (Fig. 4). The side chains of the mutated residues at position 66 could all be modeled, although the quality of the electron density maps was not as high as for the wild type. Electron density was visible for W66F in both protein chains of the asymmetric unit, but mainly in the 2Fo-Fc omit map. The W66F side chain was oriented similarly to that of the wild type tryptophan, which is in line with the similarly high Cbi binding affinities of these two BtuF variants. In contrast to W66 and W66F, the leucine side chain of the W66L variant was pointing out of the substrate-binding pocket, reminiscent of the W66 rotamer in the Cbl-bound BtuF structure. Clear density for the leucine side chain was observed in the Fo-Fc omit map for both BtuF W66L chains in the asymmetric unit. A stretch of continuous extra density was present near the corrin ring, where the tryptophan and phenylalanine side chains were found in the wild type or W66F variant of BtuF. This extra density likely originated from a bound buffer component and was modeled as a glycerol molecule that may have entered this cavity during purification or crystal cryoprotection. In the structure of the W66Y variant, density in the 2Fo-Fc and Fo-Fc omit maps allowed clear modeling of the tyrosine side chain in chain A of the asymmetric unit. Interestingly, the tyrosine side chain of W66Y was also pointing out of the substrate-binding pocket, similar to the W66L structure. In chain B, the side chain was not visible, suggesting flexibility. Extra density was also present near the corrin ring and was modeled as a glycerol molecule, similar to the W66L structure. The side chain orientation of W66L and W66Y can explain the lower Cbi binding affinities of the corresponding BtuF constructs, because these two residues do not seem to contribute to Cbi binding.

The corrin ring of Cbi can bind cyanide either at the α- or β-position. Judged by the red color of the crystals, BtuF-bound Cbi contained only one cyanide group (the di-cyano-form would have been purple). However, it could not be determined unambiguously whether cyanide was bound in the α- or β-position in all BtuF variants. Only for the W66F variant cyanide could be clearly placed at the β-position in both protein chains of the asymmetric unit. In chain A of the wild type structure, cyanide was in the α-position of Cbi. In chain B, there was some density both at the α- and β-positions, and we therefore modeled a cyanide group on both sides, each with an occupancy of 0.5. For chain A of the W66Y variant, and for chain B of W66L, bound cyanide was observed in the β-position, while there was no electron density at the α-position for the second axial ligand. In the other protein chain cyanide could be bound at either position, and therefore again modeled with an occupancy of 0.5. Thus, the BtuF substrate binding site does not seem to have a preference for the α- or β-mono-cyano form of the substrate, since the cyanide group could not be assigned to one side of the corrin ring consistently. The corrin ring nevertheless was always bound with the α-side facing the N-lobe and the β-side facing the C-lobe of BtuF.

### *In vitro* Cbl and Cbi transport by BtuCD with BtuF W66 mutants

**Figure 5:**
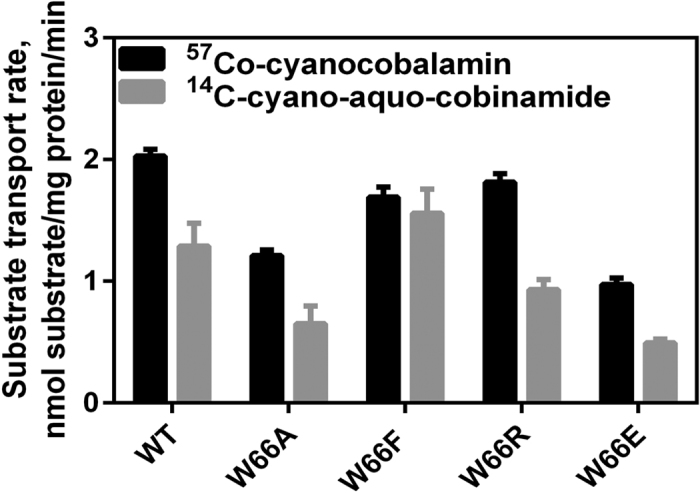
Substrate transport with BtuF W66 mutants and wild type BtuCD. Shown are the initial transport rates of Cbl and Cbi. Transport was measured under the following conditions: 4 mg/ml proteoliposomes (~0.5 μM total BtuCD), 1 μM BtuF, 2 mM ATP, 15 μM cobinamide or cyanocobalamin. Indicated are mean and S.E.M. of the transport rates of Cbl (n = 6 for wt, n = 9 for mt) or Cbi (n = 6). The graphs used to determine the initial rates are illustrated in Supplementary Figure 5.

Substrate transport was measured using commercially available ^57^Co-Cbl and in-house synthesized ^14^CN-Cbi as described above. To examine the role of W66 in substrate delivery and transport, three mutants with impaired Cbi binding (W66A, W66R, and W66E) and one with high binding affinity (W66F) were used for transport assays. Despite having lower Cbi binding affinities, Cbi transport was hardly affected by W66X substitution (Fig. 5). This finding indicates that under the conditions used, W66 is important for high affinity Cbi binding, but not for substrate delivery or transport.

## Discussion

We have developed a method to synthesize radiolabelled Cbi and have established an *in vitro* transport assay that confirms that the *E. coli* BtuCD-F binds and transports the Cbl precursor Cbi. Wild type BtuCD-F transports Cbi approximately 1.5-fold slower than Cbl, which illustrates that the size of the substrate does not inversely correlate with the rate of transport. Instead, interaction between the substrate and the translocation pathway between the BtuC subunits might affect the transport rate.

To elucidate the molecular basis of the Cbi versus Cbl specificity of BtuF, we determined the X-ray structure of Cbi-bound BtuF. A single residue, W66, was found to have a pivotal role. This residue is located in the binding site and appears to replace and thus compensate for the missing DMB group when Cbi is bound. It does so by changing its side chain rotamer to point towards the inside of the substrate binding pocket. A mutagenesis study of W66 showed that the presence of a big hydrophobic and aromatic side chain at this position is crucial for high affinity Cbi binding. In contrast, Cbl binding was much less influenced by the chemical properties of this residue, which is in line with the observed structure of W66 in Cbl-bound BtuF, where the indol moiety points away from the substrate binding pocket. Intriguingly, MD simulations had earlier suggested that the BtuF segment including W66 may act as a gate in the substrate binding and unbinding process of BtuF^17^. Our observations are in agreement with this interpretation, but single out W66 as the key residue adapting its conformation to facilitate Cbi binding.

Despite the lower binding affinities of certain W66 mutants for Cbi, substrate transport was not influenced by W66 substitution. This indicates that W66 is not involved in substrate delivery during a productive transport cycle. The transport assays were performed with a substrate concentration at which the BtuF binding pocket was fully occupied. This was crucial to prevent the binding of apo-BtuF to BtuCD, which would reduce the observed transport rate below the detection limit. Because of this experimental limitation, it was impossible to perform transport assays at lower Cbi concentrations. Nevertheless, our data suggest that accepting Cbi or Cbl as substrates requires a conformational change in BtuF, whereas the transmembrane BtuC_2_ component appears inert to the chemical differences of Cbi versus Cbl.

Analogous to BtuF, FhuD is another class III periplasmic substrate binding protein^18^,^19^,^20^, for which multiple substrate specificity has been described. FhuD is part of the iron-siderophore (ferric hydroxamate) transport system responsible for substrate delivery to the ABC transporter FhuBC. The crystal structure of gallichrome-bound FhuD identified R84 and Y106 as the two key residues involved in direct hydrogen bonding with the three hydroxamate moieties of the ligand^21^. The main interaction of gallichrome with FhuD is mediated by the hydroxamate groups and a large part of the ligand is solvent exposed. This explains why other hydroxamates such as coprogen, Desferal, or the antibiotic albomycin are also bound by FhuD. The crystal structures revealed that albomycin is bound in a similar way to gallichrome, but coprogen binding involves Y275 for hydrogen bonding to the hydroxamate moiety^22^. The most drastic change in coprogen-bound FhuD is the reorientation of W217 to allow the insertion of part of the ligand, reminiscent of W66 in BtuF.

Multiple substrate specificity has also been observed for other periplasmic substrate binding proteins. For example, DEBP (L-aspartate/L-glutamate binding protein) from *Shigella flexneri* can bind both aspartate and glutamate while simultaneously discriminating against glutamine and asparagine^23^. In this case, the side chain carboxylate of the ligand interacts with the side chains of two arginines, a serine and a histidine in the substrate binding site. It was speculated that conformational rearrangements of these residues are involved in adapting to the smaller substrate aspartate, which is analogous to the conformational rearrangement we observed in W66 of BtuF when bound to Cbi.

Poly-specificity has also been reported for components of the eukaryotic Cbl uptake system. Haptocorrin (HC), a human corrinoid binding protein, binds Cbl, Cbi, and other Cbl analogs with similarly high affinities^24^. Three bulky residues in the substrate binding site (an arginine, a tryptophan and a tyrosine) are important for Cbi binding to HC and indirectly compensate for the “missing” DMB moiety^24^,^25^. Specifically, the Cα atom of R357 moves ~2 Å towards the corrin ring and its side chain reorients towards the e-propionamide group of Cbi, thus stabilizing it in an alternate conformation via hydrogen bonding. This occurs on the same side where the DMB of bound Cbl would be located and has a space filling function similar to W66 in BtuF.

The substrate specificity of the maltose/maltodextrin uptake system, MalEFGK_2_, was also extensively studied. MalE, the periplasmic SBP, can bind maltose, maltotriose, and larger glycans up to maltodecapentaose (maltodextrin with n = 15), as well as cyclic glycans, all with similarly high affinities^26^,^27^. Structures of MalE bound to maltose, maltotriose and maltotetraose revealed that the solvent accessible amount of the substrate increases with substrate size^28^. The first three glucose units are buried in the substrate binding groove, while additional units are exposed to the surface. Nevertheless, the binding specificity of MalE does not fully correlate with the transport rates. MalFGK_2_ translocates glycans up to maltoheptaose, but the transport rates decrease with increasing substrate size. Moreover, cyclic substrates are not transported even though they are bound by MalE^29^,^30^. In this case, both the SBP and the transporter contribute to substrate specificity.

The glutamine/asparagine transporter GlnPQ has a different approach to enable dual substrate specificity. Instead of using a single, poly-specific SBP, two structurally distinct substrate binding domains (SBD1 and SBD2) are tandem-linked to the N-terminus of one transmembrane domain^31^. SBD2 has a much higher affinity for glutamine than SBD1, which in addition to glutamine binds asparagine. Three non-conserved residues are responsible for the different affinities for glutamine, although they are not directly involved in substrate binding. They presumably have a crucial role in full closure of the substrate binding site and thus lead to tighter ligand binding^32^. Furthermore, the size of the amino acid substrate itself affects opening and closure of the substrate binding site.

It is currently unknown whether other Cbl analogues can serve as substrates for BtuCD-F. However, our findings not only establish Cbi as a bona fide substrate of BtuCD-F, but offer opportunities to investigate the translocation pathway of this transporter by structural and biochemical studies, because not only can the two axial positions of the corrin ring of Cbi be modified with probes, but Cbi itself is smaller and can interact with histidine residues that might trap the substrates in the translocation pathway of BtuCD.

## Materials and Methods

### Generation of radiolabeled cobinamide

Cob(II)inamide was synthesized using a previously described method^33^ and ^14^C-cyanide-labeled through an oxidative reaction with K^14^CN (Perkin Elmer NEC079H001MC, 1 mCi) generating ^14^C-cyano-aquo-cobinamide. The mono-cyano form was preferred over the di-cyano form due to higher stability in aqueous solution and thus facilitating unambiguous quantification. A 0.85 mol equivalent of K^14^CN was added in multiple steps to an aqueous solution containing 4.8 μmol aquo-cob(II)inamide at 22 °C. The conversion of cob(II)inamide into ^14^C-cyano-aquo-cobinamide was followed by recording the UV/Vis spectrum and monitoring TLC migration pattern. The mixture was applied to reverse phase (C18) chromatography, equilibrated and washed with ddH_2_O, and products were eluted with solvents of decreasing polarity: 5:5 MeOH:H_2_O, 7:3 MeOH:H_2_O, 100% MeOH, 100% EtOH, and 100% 2-Propanol. Fractions were collected and the ^14^C-labeled substrate concentration was determined by using K^14^CN stock dilutions as calibration curve. Products in the 5:5 MeOH:H_2_O eluent were used for transport assays and stored at −20 °C.

### BtuCD purification and reconstitution into liposomes

BtuCD was expressed, purified and reconstituted as described previously with some modifications regarding buffer composition and reconstitution procedure^6^,^34^. In brief, BtuCD with an N-terminal His10-tag on BtuC was purified by IMAC and desalted into a buffer of the following composition: 50 mM Tris 7.5, 500 mm NaCl, 0.5 mM EDTA-NaOH 8 and 0.1% w/v LDAO. Purified protein was used directly for reconstitution into liposomes consisting of *E. coli* Polar lipids and α-Phosphatidylcholine (3:1 weight ratio, Avanti Polar Lipids 100600 C and 840051 C). Liposomes were destabilized with 0.3% w/v Triton X-100 for 20 min at RT. Protein was added with a ratio of 50:1 LPR and incubated for 90 min at RT. Detergent was removed by BioBeads (40 mg/ml mixture) in 4 steps and proteoliposomes were stored at −80 °C until the day of experiment. The protein concentration within the liposomes was determined by Amido black protein assay using BSA for the calibration curve.

The amount of right-side-in oriented BtuCD in liposomes was determined as described previously for ABC type II importer HmuUV with minor modifications^35^. Briefly, liposome-reconstituted BtuCD was incubated with 1 μM fluorescently labeled BtuF (BtuF_fluo_) for 30 min at RT followed by a spin to remove excess BtuF_fluo_. The pellet was washed and resuspended at 1.25 mg/ml liposome concentration. The amount of BtuF_fluo_ associated with the liposomes was determined by measuring the fluorescence signal at an excitation wavelength of 485 nm and an emission wavelength of 516 nm. Unspecific BtuF_fluo_ interaction with liposomes was measured using empty liposomes.

### Substrate transport assay with liposome reconstituted BtuCD

For the substrate transport assays radiolabeled ^14^C-cyano-aquo-cobinamide (^14^CN-Cbi, labeled in house) or ^57^Co-cyanocobalamin (^57^Co-Cbl, MP Biomedicals 06B-430000, 10.5 uCi) was used. Prior to reaction start substrate-loaded BtuF and BtuCD containing liposomes were incubated separately for 30 min to equilibrate to 22 °C. To start the transport reaction substrate-loaded BtuF was added to the BtuCD containing liposomes with final concentrations of 15 μM substrate, 1 μM BtuF and 4 mg/ml liposomes (~0.5 μM BtuCD). An ATP-regenerating system was incorporated into the liposome lumen to keep a constant ATP concentration within the time course of the experiment. Initial luminal ATP concentration was 2 mM. The transport reaction was stopped by diluting 50 μl samples in 300 μl ice cold stop buffer with the following composition: 20 mM Tris 7.5, 200 mM NaCl, 5 mM MgCl_2_, 8% w/v PEG6000 and 100 μM unlabeled Cbl. Samples were transferred to a manifold filtration system (Millipore MSFBN6B) and washed with 2 × 200 μl cold stop buffer. The radioactivity trapped on the filters was measured either by a β counter using LCS in case of ^14^CN-Cbi or a γ counter in case of ^57^Co-Cbl. Initial transport rates were determined by using linear regression in PrismGraphpad.

### Purification and crystallization of Cbi-bound BtuF

BtuF purification was performed as described previously with some modifications including His6-tag cleavage and periplasmic preparation in case of BtuF W66X mutants^9^,^10^. Additional purification steps using preparative size exclusion chromatography were performed to obtain purer sample. Final buffer composition was 10 mM Tris pH 8 and 100 mM NaCl. For crystallization purposes BtuF was concentrated in the presence of 0.2 mM di-cyano-cobinamide (Sigma C3021). Protein was supplemented with ~1 mM di-cyano-cobinamide prior to setting up crystallization trays at ~20 mg/ml. The crystallization experiment was performed by sitting drop vapor diffusion technique and incubated at 20 °C. Depending on the pH of the reservoir condition, HEPES pH 7 or Tris pH 8.2 and higher, the crystallization drops appeared orange-red or purple corresponding to the mono-cyano- or di-cyano-form of cobinamide, respectively. This observation was made despite the fact that BtuF was co-crystallized with di-cyano-cobinamide when setting up the crystallization drops. Initial wild type crystals were obtained after 3 days, while the mutant crystals grew within 1–2 weeks in optimized screens. Initial crystallization conditions were from PEG/ION Hampton screen condition H11 (1% w/v Tryptone, 50 mM HEPES sodium pH 7.0 and 12% w/v Polyethylene glycol 3350) and were optimized in 24-well plates. Orange-red rod-shaped crystals grew to ~300 microns in size and were cryoprotected by stepwise increase of glycerol to 25% v/v. Mono-cyano-cobinamide was bound in the crystal structures corresponding to the drop and crystal colors.

Data collection was performed at the Swiss Light Source (PSI, Villigen) and data processing was done with XDS^36^. Phasing of the wild type data set was performed by molecular replacement in Phenix using the Cbl-bound BtuF structure (PDB ID 1N2Z) devoid of Cbl and waters as search model. Initial phases for the Cbi-bound BtuF W66X mutants were obtained using the wild type structure with W66A and deleted waters. Iterative cycles of refinement and model building were performed with Phenix and Coot^37^,^38^. Additional refinement steps were done with Refmac5 in the CCP4 program suite. PDB file modifications like removing Hydrogens, ANISO records or alternative conformations were performed using the CCP4 program suite^39^. Final figures were prepared using PyMol (The PyMOL Molecular Graphics System, Version 1.8 Schrödinger, LLC).

### Purification of BtuF for fluorescein labeling (BtuF_fluo_) and substrate transport assays

For binding assays BtuF was labeled at a cysteine residue in the backbone helix with fluorescein-5-maleimide (Thermo scientific 62245) as described previously^11^. In brief, 50 to 100 μM BtuF Q145C (Invitrogen QuickChange) was labeled with a 10-fold molar excess of fluorescein-5-maleimide (BtuF_fluo_). The reaction was run over night at 4 °C in the presence of 1 mM TCEP to prevent oxidation and stopped with 5 mM β-mercaptoethanol. Excess label was removed by desalting with PD10 columns and the protein was further purified by size exclusion chromatography. Protein concentration was determined by Bradford assay using BSA for calibration and the labeling efficiency was determined by measuring the absorbance at 494 nm. The labeled protein was flash frozen in liquid nitrogen and stored at −80 °C until the day of use. Final storage buffer was 50 mM HEPES 7, 200 mM NaCl, 0.5 mM EDTA-NaOH 8 and 10% v/v Glycerol.

The BtuF constructs used for substrate transport assays were lacking a 3 C cleavage site, thus the His6-tag remained attached. The protein was purified and desalted into 50 mM Tris 7.5, 200 mM NaCl, 0.5 mM EDTA-NaOH 8 and 10% v/v Glycerol and stored at −80 °C until the day of experiment.

### Substrate binding assays with BtuF_fluo_

The assay is based on monitoring the ligand-induced change in emission of fluorescein attached to BtuF (BtuF_fluo_). A similar approach has been used to determine the affinity of MBP for maltose in the presence of various sAB^40^,^41^,^42^. BtuF_fluo_ at 5 nM was incubated with a range of substrate concentrations from 0.02 nM to 33 μM. After incubation for 30 min at 10 °C the fluorescence intensity was recorded at an excitation wavelength of 485 nm and emission at 528 nm. The maximum signal was recorded in the absence of substrate and decreased with increasing substrate concentration. The difference of the maximum intensity minus the signal at a certain substrate concentration was plotted against the substrate concentration. One site –specific binding in Prism GraphPad was used to fit the data for K_D_ determination (GraphPad Prism version 6.04 for Windows, GraphPad Software, La Jolla California USA, www.graphpad.com”). Binding buffer composition was 50 mM Tris 7.5 and 200 mM NaCl.

## Additional Information

**Accession codes:** Structure factors and coordinates are deposited in the Protein Data Bank under the following accession codes: 5M29 (BtuF-Cbi), 5M2Q (BtuF_W66F_-Cbi), 5M34 (BtuF_W66Y_-Cbi) and 5M3B (BtuF_W66L_-Cbi).

**How to cite this article**: Mireku, S. A. *et al*. Conformational Change of a Tryptophan Residue in BtuF Facilitates Binding and Transport of Cobinamide by the Vitamin B12 Transporter BtuCD-F. *Sci. Rep.* **7**, 41575; doi: 10.1038/srep41575 (2017).

**Publisher's note:** Springer Nature remains neutral with regard to jurisdictional claims in published maps and institutional affiliations.

## Supplementary Material

Supplementary Information

## Figures and Tables

**Table 1:** Dissociation constant K_D_ values of Cbl and Cbi binding to wild type BtuF_fluo_ and W66 mutants.

E.c. BtuF	K_D_ of Cbl, nM	K_D_ of Cbi, nM or μM
Wild type	9.1 ± 2.6	40 ± 10 nM
W66F	8.0 ± 1.9	30 ± 7 nM
W66Y	5.7 ± 1.7	0.15 ± 0.03 μM
W66L	15 ± 2	0.12 ± 0.02 μM
W66H	5.5 ± 1.7	0.5 ± 0.1 μM
W66R	68 ± 13	0.8 ± 0.2 μM
W66A	14 ± 4	1.8 ± 0.8 μM
W66E	57 ± 15	2.9 ± 0.8 μM

The substrate binding assay is based on fluorescence quenching of fluorescein labeled BtuF (BtuF_fluo_) in a substrate concentration dependent manner. BtuF_fluo_ was used at 5 nM and substrate concentration was varied from 0.02 nM to 33 μM. Note that K_D_ values are represented in nM or μM and indicated are mean and S.E.M. (n = 12, for W66R n = 9). Example curves used to determine the K_D_ values are shown in Fig. 3 and Supplementary Figure 4.

**Table 2 t2:** Crystallographic table with data collection and refinement statistics.

	BtuF-Cbi	BtuF_W66F_-Cbi	BtuF_W66Y_-Cbi	BtuF_W66L_-Cbi
**Data collection**				
Wavelength (Å)	1.0000	0.9779	0.9779	0.9779
Resolution range (Å)	26.7–1.5	19.7–1.7	26.7–1.6	19.7–1.5
Space group	C2	C2	C 2	C 2
Unit cell dimensions				
a, b, c (Å)	138.15 90.56 50.84	136.56 90.67 50.81	139.83 90.8 51.07	136.28 90.46 50.81
α, β, γ (°)	90 110.90 90	90 110.87 90	90 111.25 90	90 110.74 90
Total reflections	316217 (29743)	188811 (18658)	208631 (20239)	271108 (26105)
Unique reflections	89627 (8616)	61184 (6145)	73585 (7648)	89398 (8823)
Multiplicity	3.5 (3.4)	3.1 (3.0)	2.8 (2.8)	3.0 (2.9)
Completeness (%)	96 (93)	96 (97)	99 (95)	97 (97)
Mean I/sigma(I)	10.21 (1.26)	10.89 (1.39)	9.77 (1.56)	11.00 (1.53)
Wilson B-factor (Å^2^)	23.05	25.01	25.55	20.22
*R*_*merge*_ (%)	5.9 (84.9)	7.2 (81.6)	6.4 (66.5)	6.0 (61.0)
*R*_*meas*_ (%)	7.0 (100.4)	8.7 (99.8)	7.8 (81.8)	7.3 (74.3)
CC _½_ (%)	99.8 (74.8)	99.7 (64.7)	99.7 (65.3)	99.8 (59.3)
**Refinement**				
Reflections used in refinement	89615 (8600)	61168 (6125)	77398 (7648)	89061 (8823)
Reflections used for R-free	4477 (430)	3055 (306)	3827 (362)	4452 (442)
*R*_*work*_ (%)	17.9 (47.4)	20.9 (46.7)	19.5 (39.9)	19.6 (32.5)
*R*_*free*_ *(5% of data)* (%)	21.3 (45.9)	24.9 (45.5)	23.7 (41.3)	22.9 (33.9)
No of non-hydrogen atoms	4275	4086	4146	4268
Macromolecules	3583	3538	3538	3547
Ligands	160	158	158	166
Water	532	390	450	555
Protein residues	463	460	461	463
RMS bonds (Å)	0.020	0.020	0.049	0.055
RMS angles (°)	1.82	1.93	2.34	2.02
Ramachandran favored (%)	97	98	96	97
Ramachandran allowed (%)	2.4	2.0	3.1	2.0
Ramachandran outliers (%)	0.44	0.22	0.67	0.67
Rotamer outliers (%)	1.8	2.9	4.7	1.0
Clashscore	5.6	8.6	12.48	6.7
Average B-factor (Å^2^)	32.11	34.36	33.62	25.70
Macromolecules	30.49	33.67	32.68	23.87
Ligands	32.40	32.96	34.57	28.28
Solvent	42.94	41.23	40.65	36.62
**PDB ID**	5M29	5M2Q	5M34	5M3B

Highest-resolution shell values are shown in parentheses. The correlation coefficient is abbreviated CC^43^.
