# Supplementary material for: Conformational Change of a Tryptophan Residue in BtuF Facilitates Binding and Transport of Cobinamide by the Vitamin B12 Transporter BtuCD-F
Source: Sci Rep. 2017 Jan 27;7:41575. doi: 10.1038/srep41575 (PMC5269720; doi:10.1038/srep41575)
Supplement: Supplementary Information [file srep41575-s1.pdf]

Conformational Change of a Tryptophan Residue in BtuF Facilitates Binding and Transport of Cobinamide by the Vitamin B12 Transporter BtuCD-F

Mireku SA[$], Ruetz M[‡], Zhou T[$], Korkhov VM[$,*], Kraeutler B[‡] and Locher KP[$]

Supplementary information

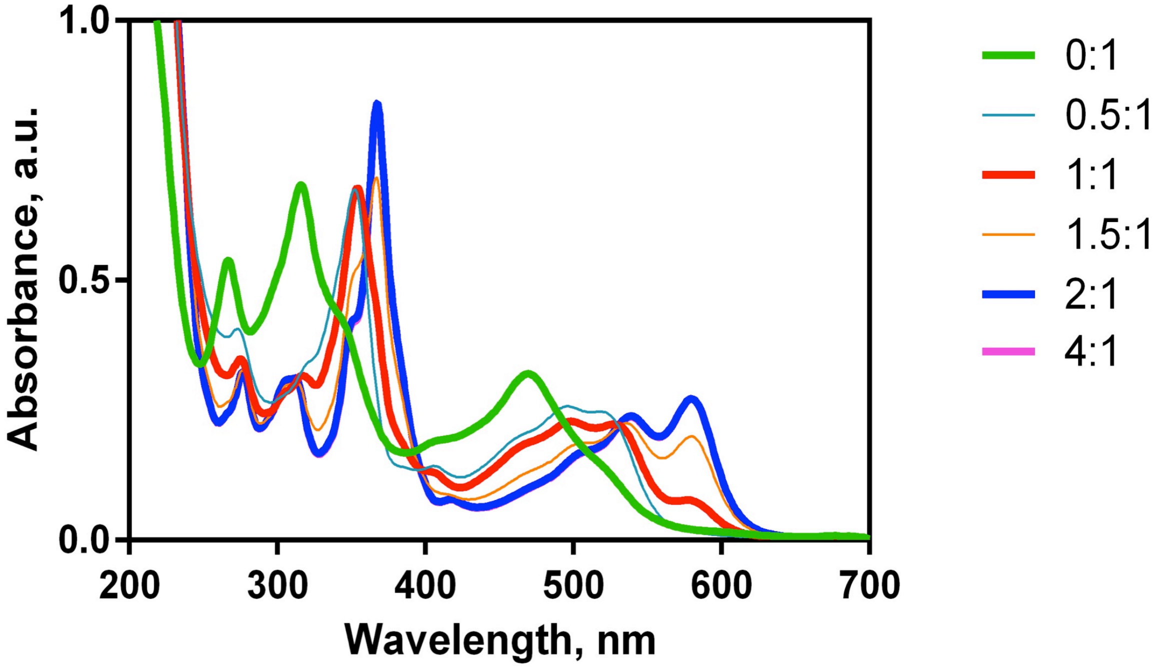

Supplementary figure 1 UV/Vis spectra of mono- and di-cyano-cobinamide. KCN was titrated to 41nmol aquo-cob(II)inamide using 0, 0.5, 1, 1.5, 2 and 4 mol equivalent of CN (CN to cob(II) ratios 0:1, 0.5:1, 1:1, 1.5:1, 2:1 and 4:1). UV/Vis spectra were recorded 30min after incubation and at a substrate concentration of 41 µM. The initial substrate aquo-cob(II)inamide is shown in green and the mono- and di-cyano-forms of the product are represented in red and blue, respectively. Di-cyano-cobinamide shows an additional peak around 580 nm, which is absent in the spectrum of aquo-cob(II)inamide or the mono-cyano-form. Note that at a 1:1 mol ratio di-cyano-cobinamide formation starts to occur.

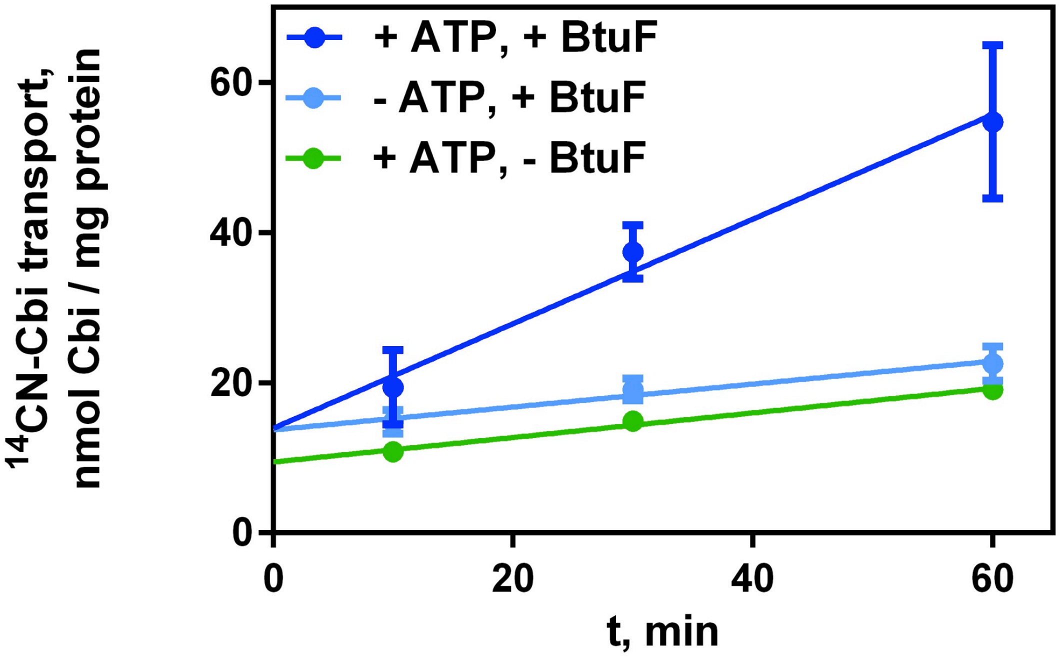

Supplementary figure 2 ***In vitro*** **Cbi transport by liposome reconstituted BtuCD.** Transport was measured under the following conditions: 4mg/ml proteoliposomes (~0.5 µM total BtuCD), 1 µM BtuF, 15 µM cobinamide and 2 mM ATP. Unspecific radioligand binding to liposomes was determined by omission of ATP or BtuF. Shown are mean ± S.E.M (n=6) and for calculated transport rates see figure 1D.

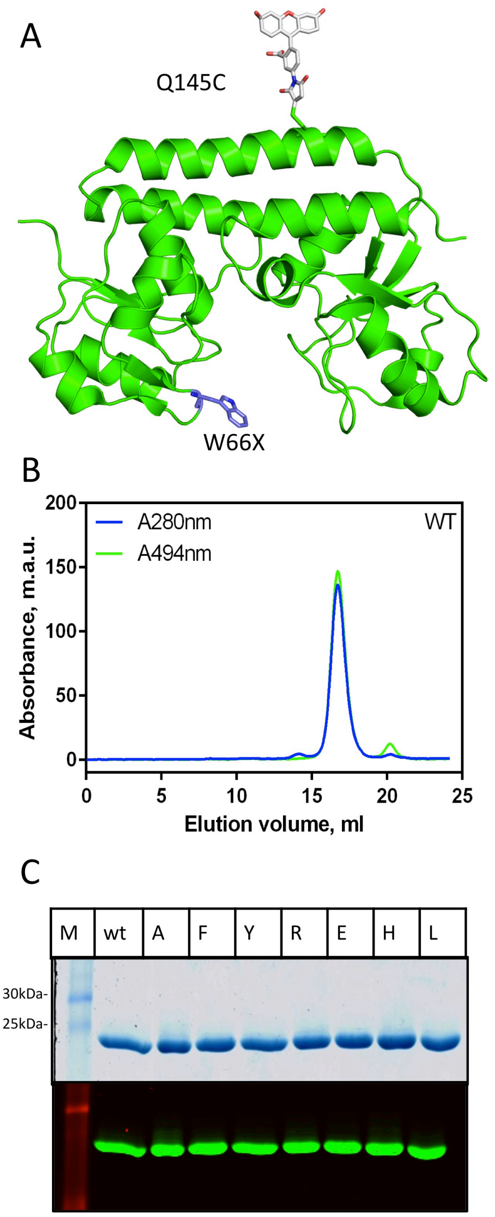

Supplementary figure 3 Purification and fluorescein-5-maleimide labeling of BtuF W66 mutants. A) Model of fluorescein labeled BtuF (BtuF$_{fluo}$). Indicated are the residues used for BtuF labeling with fluorescein-5-maleimide (grey sticks), Q145C in the backbone helix, and the site of mutagenesis, W66X in the substrate binding site (violet sticks) (PDB ID 1N2Z). **B**) Preparative size exclusion chromatography profile of purified BtuF$_{fluo}$ showed a monodispersed peak. Protein was monitored at 280 nm and fluorescein-5-maleimide at 494 nm. Shown is the profile for wild type BtuF, similar traces were obtained for the mutants. **C**) Coomassie stained 15 % non-reducing SDS PAGE of wild type BtuF and mutants (W66X, X = A / F / Y / R / E / H / L) showed a clean purification. In gel fluorescence was detected at an excitation wavelength of 488 nm and an emission wavelength of 526 nm.

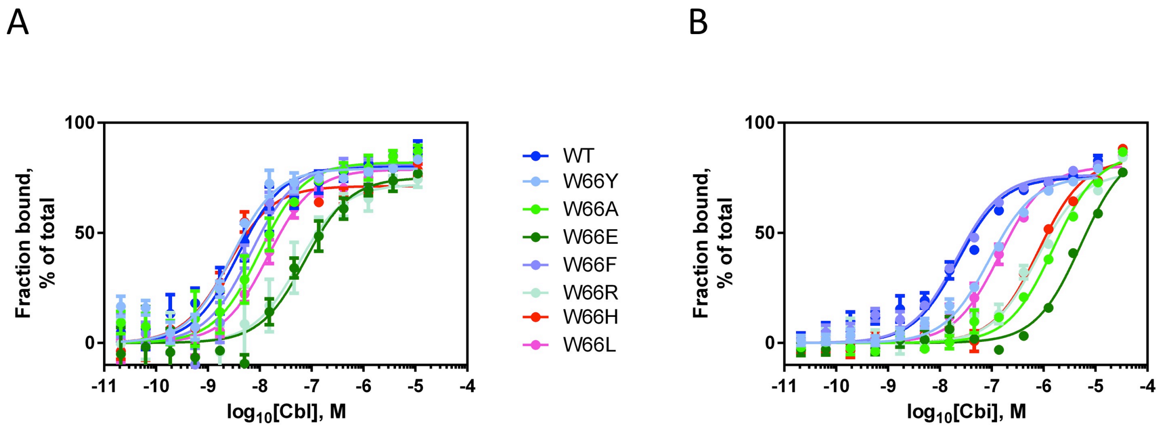

Supplementary figure 4 Cbl and Cbi binding by wild type BtuF$_{fluo}$ and W66 mutants. Substrate binding assay based on fluorescence quenching of fluorescein labeled BtuF (BtuF$_{fluo}$) in a substrate concentration dependent manner. BtuF$_{fluo}$ was used at 5 nM and substrate concentration was varied from 0.02 nM to 33 µM. Shown is an example of one experiment performed in triplicates to determine the substrate affinities. Panel **A** shows the binding curves for Cbl and panel **B** the ones for Cbi. The same color coding was used in B as indicated in A. Note the logarithmic scale of the x-axis. Indicated are mean ± SD for n=3.

A

B

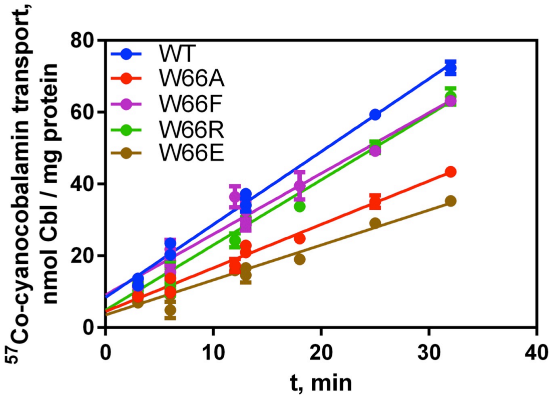

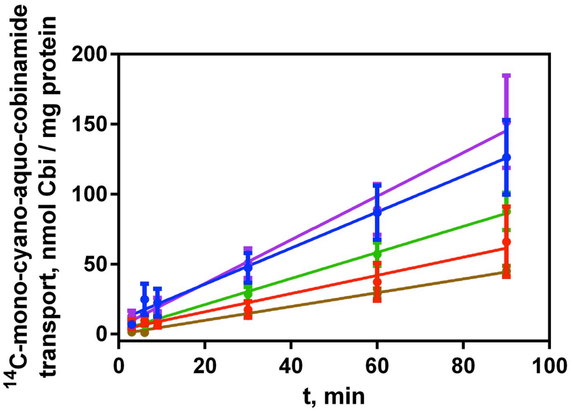

Supplementary figure 5 *In vitro* Cbl and Cbi transport with BtuF W66 mutants and wild type BtuCD. Illustration of the lines used to determine the initial transport rates indicated in figure 5. Panel **A** represents Cbl and panel **B** Cbi transport using the same color coding. Transport was measured under the following conditions: 4 mg/ml proteoliposomes (~0.5 μM total BtuCD), 1 μM BtuF, 15 μM cobinamide or cyanocobalamin and 2 mM ATP. Unspecific radioligand binding to liposomes was determined by omission of ATP and subtracted from the transport data. Shown are mean and S.E.M for Cbl (n=6 for wt, n=9 for mt) and Cbi (n=6) transport.
